# Effect of Chain Extending Cross-Linkers on the Disintegration Behavior of Composted PBAT/PLA Blown Films

**DOI:** 10.3390/ijms24054525

**Published:** 2023-02-24

**Authors:** Juliana V. C. Azevedo, Berenika Hausnerova, Bernhard Möginger, Tomas Sopik

**Affiliations:** 1 Faculty of Technology, Tomas Bata University in Zlín, Vavreckova 275, 76001 Zlín, Czech Republic; 2 Department of Natural Sciences, University of Applied Sciences Bonn-Rhein-Sieg, von Liebig Str. 20, 53359 Rheinbach, Germany; 3 BIO-FED, Branch of AKRO-PLASTIC GmbH, BioCampus Cologne, Nattermannallee 1, 50829 Köln, Germany; 4 Centre of Polymer Systems, University Institute, Tomas Bata University in Zlín, Nam. T.G. Masaryka 5555, 76001 Zlín, Czech Republic

**Keywords:** poly(butylene adipate terephthalate), poly(lactic acid), blown film, chain extending cross-linker, degree of disintegration, disintegration kinetics, molecular mass degradation

## Abstract

A biodegradable blend of PBAT—poly(butylene adipate-co-terephthalate)—and PLA—poly(lactic acid)—for blown film extrusion was modified with four multi-functional chain extending cross-linkers (CECL). The anisotropic morphology introduced during film blowing affects the degradation processes. Given that two CECL increased the melt flow rate (MFR) of tris(2,4-di-tert-butylphenyl)phosphite (V1) and 1,3-phenylenebisoxazoline (V2) and the other two reduced it (aromatic polycarbodiimide (V3) and poly(4,4-dicyclohexylmethanecarbodiimide) (V4)), their compost (bio-)disintegration behavior was investigated. It was significantly altered with respect to the unmodified reference blend (REF). The disintegration behavior at 30 and 60 °C was investigated by determining changes in mass, Young’s moduli, tensile strengths, elongations at break and thermal properties. In order to quantify the disintegration behavior, the hole areas of blown films were evaluated after compost storage at 60 °C to calculate the kinetics of the time dependent degrees of disintegration. The kinetic model of disintegration provides two parameters: initiation time and disintegration time. They quantify the effects of the CECL on the disintegration behavior of the PBAT/PLA compound. Differential scanning calorimetry (DSC) revealed a pronounced annealing effect during storage in compost at 30 °C, as well as the occurrence of an additional step-like increase in the heat flow at 75 °C after storage at 60 °C. The disintegration consists of processes which affect amorphous and crystalline phase of PBAT in different manner that cannot be understood by a hydrolytic chain degradation only. Furthermore, gel permeation chromatography (GPC) revealed molecular degradation only at 60 °C for the REF and V1 after 7 days of compost storage. The observed losses of mass and cross-sectional area seem to be attributed more to mechanical decay than to molecular degradation for the given compost storage times.

## 1. Introduction

The advances in materials sciences and technology allow for developing environmentally suitable packaging products [1,2,3]. As a response to increasing plastic use and the related environmental pollution, bioplastics have become highly interesting in the last decade [4,5,6]. The development of biodegradable polymers seems to be an effective way to partly solve the plastics waste problem. In this respect, commercially available PBAT—poly(butylene adipate-co-terephthalate)—and PLA—poly(lactic acid)—are predominantly selected by bioplastics manufacturers [7,8,9,10,11] and used for the production of blown films mainly for shoppers bags, fruit and vegetable bags, and waste bags (e.g., Ecovio^®^ by BASF).

PBAT is a random copolymer of butylene adipate and terephthalate that owes its biodegradability to the butylene adipate groups and its stability and mechanical properties to the terephthalate groups [11,12,13,14]. It is a flexible polymer with high elongation at break and good processing properties. PLA is entirely renewable if originating from a starch [15]. Due to the brittle behavior of PLA, it is inappropriate for applications requiring high deformation strains [16,17]. Therefore, it is often modified by plasticizers and chain extenders [18,19,20]. The flexibility and toughness of PBAT may compensate for the brittleness of PLA in their blends. The studies conducted on the processing of PBAT and PLA blends focus on balancing the properties required for specific applications [21,22,23].

An important factor involves denoting the experimental conditions under which biodegradation takes place in order to investigate its effects on the disintegration of PLA/PBAT blends. Weng et al. [24] investigated a 40/60 PBAT/PLA blend cast to films after granulation, which were then composted in a soil environment. DSC analysis of the pure polymers showed that the melting temperature of the PBAT was slightly decreased after degradation, while the melting temperature of the PLA was increased. The changes in the melting temperatures of the blend before and after degradation were identical to those of the corresponding polymers. However, combined thermogravimetric and elemental analyses revealed that the degradation rates of the components in the PBAT/PLA blend differed from those of the individual polymers. Nevertheless, all samples of PBAT, PLA and PBAT/PLA were fragmented after four months of degradation in soil. Scanning Electron Microscope (SEM) revealed an increased roughness on the surfaces, but the blend did not show voids.

Given that the introduction of filler and additives also affects the biodegradation behavior and kinetics of PBAT/PLA blends, due to increased diffusion paths and the possible abiotic properties of the filler systems, Tolga et al. [25] investigated the disintegration process of a PLA containing mineral fillers blended with other biodegradable polymers. For 2 mm thick plates of the 30/70 PBAT/PLA blend, they found that the disintegration process started after an initiation time of 4 weeks in a compost at 60 °C and 70% relative humidity. After 12 weeks, the degree of disintegration exceeded 25%.

Further, from the behavior of PLA/PBAT blends, one can conclude that the disintegration rates decrease with increasing filler contents during the first stage of disintegration [25]. Freitas et al. [26] studied the effect of montmorillonite clay (MMT) filler on PLA/PBAT blends that were modified with chain extenders. For unfilled PLA/PBAT blends, they found the highest emission rate of CO_2_, which indicated that the degradation rate depended on the available polymer surface. Filler particles may also hinder microorganisms from penetrating the polymer or have partly antibiotic effects. In each case, MMT reduced the available surface, which led to lower emission rates of CO_2_, and, thus, lower biodegradation rates.

Touchaleaume et al. [27] investigated 40 µm thick films of a 70/30 PBAT/PLA blend in vineyard soil for 24 months at ambient temperatures with respect to the amount of degraded surface area. They observed no degradation over 24 months, which indicated that the PBAT matrix does not allow significant degradation at ambient temperatures, although an increased surface roughness was found. This result is supported by degradation tests of a 55/45 PBAT/PLA blend for 180 days at 30 °C, in which Lamparelli et al. [28] found weight gains of 0.7% in soil and 1.4% in an aqueous medium. On the other hand, compost storage for 70 days at 60 °C [27] revealed initiation times of the degradation process of 4 to 5 days for an 18 month aged blend and of 6 days for the fresh blend. They successfully fitted their measurements (R² > 0.99) using the Hill model [29], but they did not provide any fit parameters of degradation kinetics.

From the above-mentioned investigations, one can conclude that biodegradation can occur only after certain initiation times in which the polymer chains are fractured by hydrolysis, photo-oxidation, and thermal oxidation into pieces that can be metabolized by micro-organisms [25,27,30,31]. Deeper insights into degradation modes of PBAT films are given in a review article by Liu et al. [32].

At present, an effect of multi-functional chain extending cross-linkers (CECL) on disintegration, as well as mechanical and thermal properties, have been investigated for the extrusion of blown films of PBAT/PLA blends very scarcely. Most studies were carried out on samples manufactured by other processing techniques, e.g., injection or compression molding that did not take into account the pronounced anisotropic morphology introduced by blown film extrusion. Our previous study [33] showed that the chemical reactions caused by CECL in PBAT/PLA blends were incomplete after compounding, and that the stretching during blown film extrusion brought the appropriate molecular groups into reach by promoting further cross-linking, chain scission, or other reactions. The objective of this study is to investigate in detail the effects of four CECLs on the disintegration behavior and kinetics of a PBAT/PLA blend under compost storage.

## 2. Results

**Figure 1:**
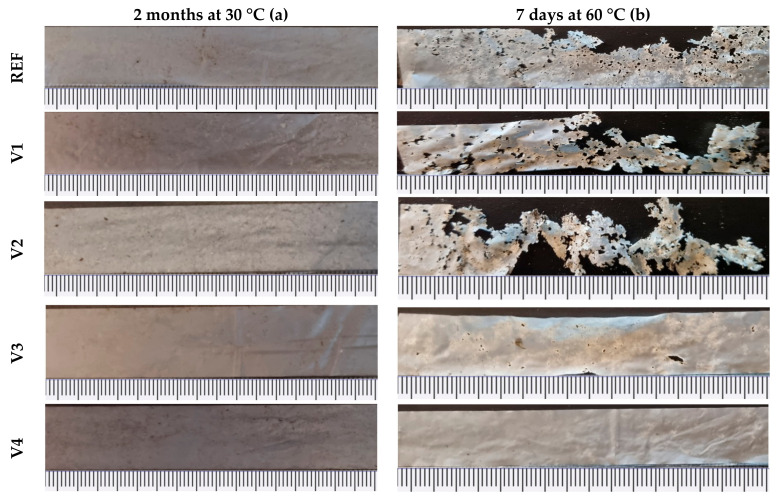
Compost storage for 2 months at 30 °C (**a**) and 7 days at 60 °C (**b**) on 25 µm-thick films of PBAT/PLA compounds; scaling in mm.

The visual inspection of the films shows that temperature had a significant effect on the disintegration behavior of the PBAT/PLA compounds modified by four CECLs (for details see Section 4.1). Figure 1 shows that, at 30 °C, the samples of REF (unmodified blend), V1 (CECL = tris(2,4-di-tert-butylphenyl) phosphite), and V2 (CECL = 1,3-phenylenebisoxazoline) exhibited small stains on the surface, which indicated a starting disintegration after 2 months. At 30 °C, PLA is in the glassy state (glass temperature of 60 °C), which prevents its degradation.

At 60 °C, the REF exhibited a mean disintegration after 7 days, and V1 and V2 exhibited severe disintegrations, whereas V3 (CECL = aromatic polycarbodiimide) showed a starting disintegration, and V4 (CECL = poly(4,4-dicyclohexylmethane carbodiimide) still seemed to be unaffected. The states of disintegration were in line with the increased melt flow ratios of V1 and V2 when compared to REF, as well as with the decreased melt flow ratios of V3 and V4 [33]. This showed that disintegration was eased in the case of shorter and more mobile polymer chains.

### 2.1. Effects of Compost Storage on Mass Change

**Figure 2:**
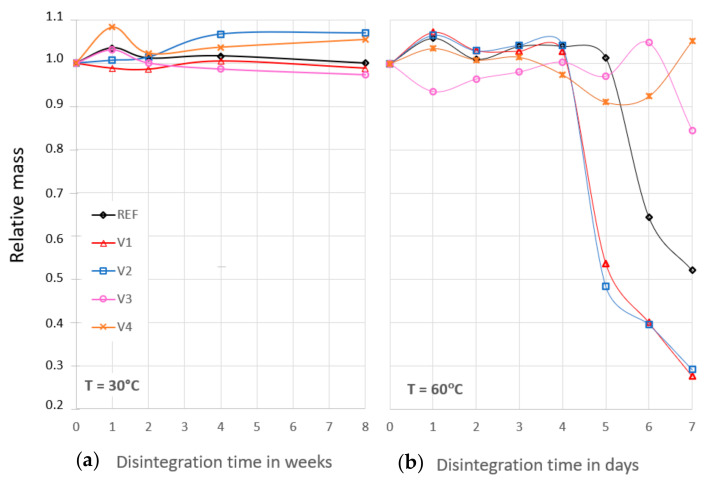
Relative mass change of 25 µm-thick films of REF and V1 to V4 over 8 weeks at 30 °C (**a**) and 7 days at 60 °C (**b**) of disintegration in compost; standard deviations (STD) are between 0.03 and 0.07, the lines serve for better visualization.

At 30 °C, the relative masses of the samples of REF and V1 to V4 remained more or less unchanged within the range of the scatter of the mass determination after compost storage for 8 weeks. Figure 2 indicates that the disintegration processes did not lead to mass decreases yet. The mass variations can be attributed to processes which occur within the initiation time, e.g., mass increase due to chemical reactions, humidity uptake, and remaining compost particles, or mass decrease due to releases of degradation reactions or loss of micro-plastic particles during cleaning.

At 60 °C, the relative masses remained unchanged only for 4 days. Figure 2 shows that, for V1 and V2, a pronounced mass decrease was consequently observed. The REF exhibited a less pronounced mass decrease between day 5 and 6. The V3 showed a tendency of decreasing mass, whereas the V4 seemed to not be disintegrated, even after 7 days. This shows that the initiation times of disintegration are significantly affected by the chosen CECL and the induced chemical changes. Furthermore, significant disintegration was observed for the REF, V1, and V2 after 7 days at 60 °C. This supports the interpretation that the stains on the REF, V1, and V2 in Figure 1 represented a beginning disintegration.

Because of the scatter of the mass measurements, the disintegration was confirmed only if the masses had decreased to less than 90% of the initial value. Thus, the time *t*_80%_ was the determined time at which the masses of the films reached 80% of their initial masses, as is shown in Figure 2, and it represents the upper limit of the initiation time.

The data indicated that there was a mass increase at the beginning of the compost storage due to the initial disintegration processes. To estimate the mass scatter due to adsorbed and biodegradation-generated low molecular weight components, the initial masses of the DSC experiments were compared to the masses after the second heating runs. The mass losses were between 2 and 12%, which showed that there was a remarkable content of low molecular components in the films, as is shown in Table 1.

After 8 weeks at 30 °C, the REF had lost 4% of its mass, and 7% of its mass after 7 days at 60 °C. The decrease in the V1 and V2 exceeded that of the REF, which showed that the biodegradation of the V1 and V2 was more pronounced. For the V3 and V4, the decreases were less than for the REF, which showed that cross-linking hindered biodegradation and decelerated disintegration. However, the decreases were still within the scatter of the relative masses during the initiation times. This is a further indication for a mass increase due to the uptake and generation of low molecular weight components during the initiation times of compost storage, in addition to the contributions of adherent compost particles.

**Figure 3:**
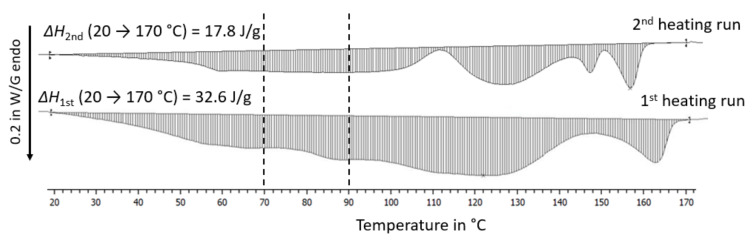
First and second heating run of REF after compost storage for 7 days at 60 °C showing the enthalpies between 20 and 170 °C as hatched areas. The dashed lines mark the range 70 to 90 °C in which most of the water desorption is expected to have occurred.

For all compounds, the DSC traces of the first run differed significantly from the second run, as is shown in Figure 3, and the enthalpies also differed between 20 and 170 °C. These differences can be attributed to the processing history (which was similar for all films) and the reached state of biodegradation. As polyamides are typically dried at 80 °C (a temperature which is also above the *T*_g_ of PLA), the enthalpies between 70 and 90 °C were determined, as is shown in Table 1. It can be seen that these enthalpies correlated qualitatively with the mass loss for the REF, V1, and V2 and that they were almost equal for the V3 and V4. Given that the content of CECLs was low, one can assume that the water contents of all films were identical. Thus, the differences in the mass losses in Table 1 have to have been generated by volatile low molecular linked to the initial processes of biodegradation.

### 2.2. Effects of Compost Storage on Disintegration Kinetics

**Figure 4:**
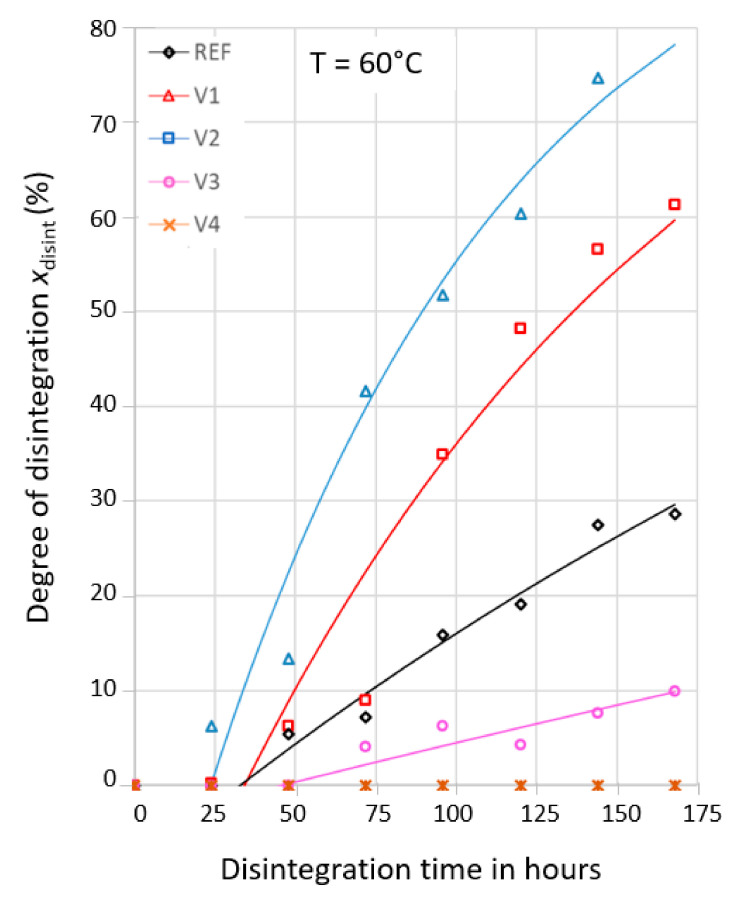
Time dependent degree of disintegration at 60 °C of REF and V1 to V4; comparison of experimental data (symbols) to fits according to Equation (8); STD from 0.03 to 0.05.

After compost storage at 60 °C, the films were further investigated with respect to their disintegration kinetics by determining the cross-sectional areas of holes to elucidate the kinetic parameters of initiation time *t*_init_ and disintegration time *τ*_disint_ for the REF and for V1 to V4. The experimental data can satisfactorily be fitted by Equation (8) with a reasonable R^2^, as are shown in Figure 4 and Table 2. In the model, a single dominating disintegration process was assumed using an approach that was used to describe the hydrolytic degradation of neat PLA, PLA modified with a carbodiimide, and PLA wood flour composites [34,35]. The smaller measured time dependent degrees of disintegration *x*_disint_ of the REF, V1, and V2 when compared to the predicted values can be explained by the fact that the disintegration had already happened prior to the first visible holes.

The times *t*_80%_ (to the mass decrease of 80%) exceeded the initiation times *t*_init_ by three to four factors, as is shown in Table 2. This shows that the initiation of disintegration depends on the considered property—mass loss or hole generation.

Obviously, some degree of disintegration has to happen prior to the appearance of the first holes. Thus, the determined initiation times *t*_init_ have to depend on the film thickness. If one assumes that the first appearance of holes depends linearly on the film thickness, the initiation time of V1 (30 µm instead of 25 µm) would be roughly 20% too long—leading to a corrected *t*_init_ ≈ 27 h. The V4 did not show any holes within 7 days, which indicated that the molecular structure generated by the CECL poly(4,4-dicyclohexylmethane carbodiimide) prevented significant disintegration.

The disintegration times *τ*_disint_ show that the CECLs significantly affected the disintegration processes. The disintegration rates of the V1 and V2 exceeded that of the REF by 2.5 times and 4 times, respectively, whereas it was decreased to a factor 0.25 for the V3. Given that the V4 did not show any holes within 7 days, it was not evaluated.

### 2.3. Effects of Compost Storage on Mechanical Properties

**Figure 5:**
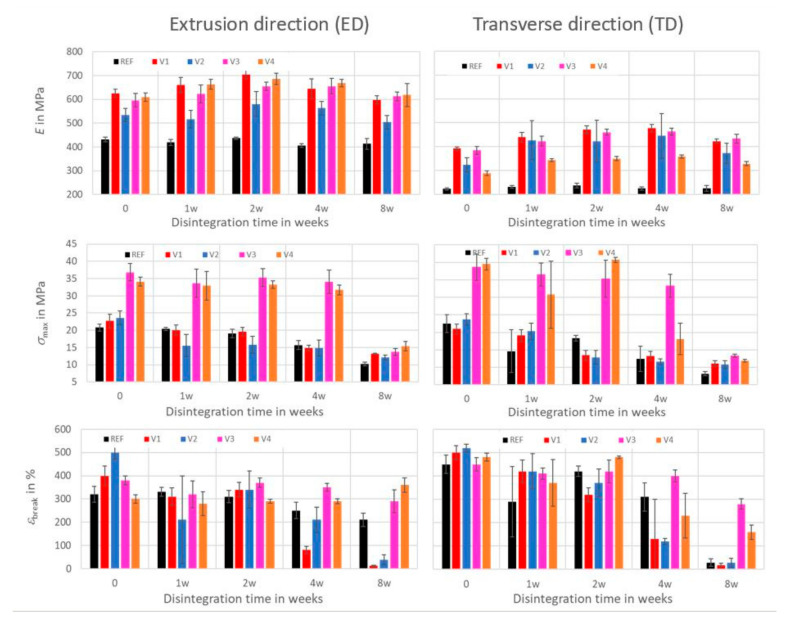
Time dependent effects of disintegration on mechanical properties (Young’s modulus *E*, tensile strength *σ*_max_, elongation at break *ɛ*_break_) in extrusion direction (ED) and transverse direction (TD) of films after storage in compost at 30 °C.

The Young’s moduli in the extrusion direction (ED) were roughly double of those in transverse direction (TD), which reflected the film anisotropy due to processing. Compost storage of the REF and V1 to V4 for 8 weeks at 30 °C initially led to an increase in the Young’s moduli in the order of 20% before they were slowly decreased again, as is shown in Figure 5. This increase can be explained by the annealing effects in the amorphous phases of PLA [36] and by the post-crystallization of PBAT. The PLA in its disperse phase was protected by the PBAT matrix against any kind of degradation and disintegration at the beginning of the compost storage at 30 °C, and annealing could happen undisturbed. Jian et al. [8] reported that PBAT starts to crystallize at 60 °C if heated for 10 °C/min during DSC. Therefore, a slow post-crystallization of amorphous PBAT can already be expected at 30 °C if humidity provides more mobility to the polymer chains. Both processes may increase stiffness. Interestingly, the maximum Young’s moduli were determined after 2 weeks in the ED, while 4 weeks were needed in the TD, which indicated that mechanical degradation happened differently in the ED and TD during initiation time. This can be understood by the fact that the films exhibit significantly different morphologies on their fracture surfaces in the ED and TD [37]. Thus, during the initiation phase, one direction can be more affected by biodegradation processes than the other.

Compost storage for 8 weeks at 30 °C led to a continuous decrease in the tensile strengths of the REF, V1, and V2 in the order of 50 to 70% in the ED and TD. The V3 and V4 exhibited a plateau of tensile strength for 4 weeks in the ED before a decrease of 60 to 70% occurred. In the TD, the V3 exhibited a plateau of tensile strength for 4 weeks, and the V4 exhibited a plateau of tensile strength for 2 weeks.

The elongations at break exhibited a plateau for 2 weeks for the REF, V1, and V2 in the ED, with subsequent decreases of 35% (REF) and almost 100% (V1 and V2). For the V3, the plateau lasted 4 weeks, followed by a decrease of 20%, whereas no decrease was observed for the V4, even after 8 weeks. In the TD, the elongations at break of the REF and V2 exhibited a plateau for 2 weeks before they decreased, whereas the V1 showed a continuous decrease. Their elongations at break after 8 weeks dropped to 5 to 30% of the initial values. The V3 and V4 exhibited plateaus of elongations at break in the TD that lasted 4 weeks and 2 weeks, respectively, with subsequent decreases of 30 and 70%, respectively.

**Figure 6:**
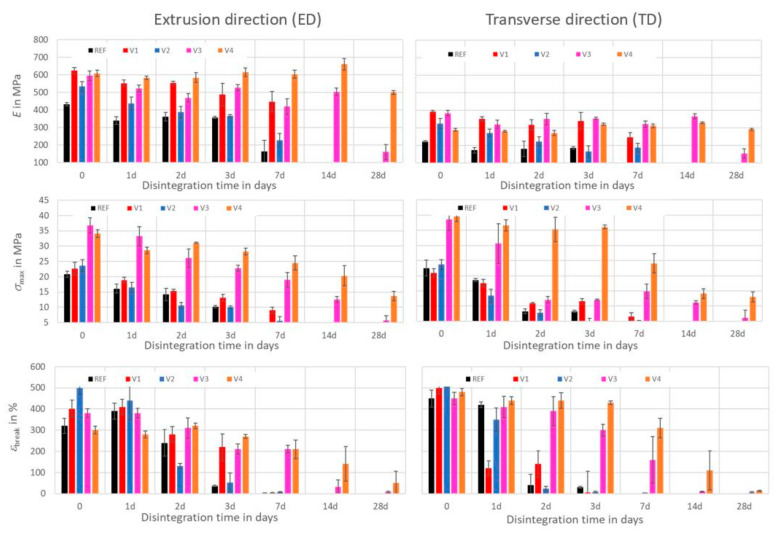
Time dependent effects of disintegration on mechanical properties (Young’s modulus *E*, tensile strength *σ*_max_, elongation at break *ɛ*_break_) in extrusion direction (ED) and transverse direction (TD) of films after storage in compost at 60 °C.

Compost storage at 60 °C, as is depicted in Figure 6, had pronounced impact on the mechanical properties of the films. Annealing effects in the amorphous phase of the PLA did not continue to occur, as the PLA was now above the glass transition temperature. Therefore, no increases in Young’s moduli were further observed. After one week, it was hardly possible to perform tensile tests with films of the REF, V1, and V2, due to severe mechanical disintegration. In the ED and TD, the Young’s moduli of the REF remained on a plateau for the first 3 days. Then, they continuously decreased close to zero within a week. The Young’s moduli of the V1 and V2 continuously decreased after 7 days to 70% and 50%, respectively, in the ED and TD. For the V3, its Young’s moduli in the ED and TD remained on a plateau for 14 days before they decreased by at least 50%. The Young’s moduli of the V4 remained on a plateau for almost 28 days. Only the modulus in the ED was 15% lower after 28 days.

In the ED and TD, the tensile strengths of the REF, V1, and V2 decreased continuously to approximately 20% of their initial values after 7 days. For the V3, its Young’s moduli in the ED decreased continuously to less than 20% after 28 days, whereas in the TD, they decreased to 30% during the first 2 days and remained on that plateau for 2 weeks before they further decreased to less than 20%. For the V4, the tensile strength in the ED decreased continuously to approximately 40% after 28 days, whereas in the TD, it remained on a plateau for 3 days before it decreased to 30%.

The elongations at break in the ED and TD of the REF remained on a plateau for 1 day and then decreased to approximately 10% of their initial values after 3 days. For the V1, a plateau was observed in the ED for 1 day with a subsequent decrease that was almost close to zero after 7 days. In the TD, the decrease close to zero was already reached after 3 days. The V2 exhibited a continuous decrease and reached elongations at break of a few percentage points after 7 days in the ED and after 3 days in the TD. The V3 exhibited a plateau for 1 day and a slow decrease to a few percentage points after 14 days in both the ED and TD. For the V4, a plateau was observed for 3 days, followed by a decrease to 50% in the ED and 10% in the TD.

Tensile strengths and elongations at break indicate a high sensitivity with respect to structural changes of the films, and they showed that the films of the REF and V1 to V4 had also been mechanically degraded during compost storage at 30 °C. Obviously, these processes were still in the state of initiation with negligible effects on film masses. At 60 °C, the degradation processes were significantly accelerated, as the REF, V1, and V2 were severely disintegrated after 7 days (see Figure 1), and both their tensile strengths and elongations at break dropped to low values.

### 2.4. Effects of Compost Storage on Thermal Properties

**Figure 7:**
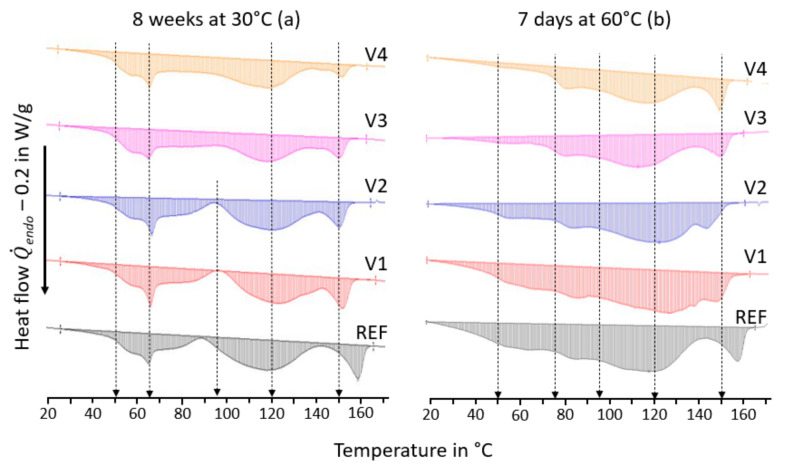
DSC traces of the first heating run of REF and V1 to V4 after compost storage for 8 weeks at 30 °C (**a**) and 7 days at 60 °C (**b**).

The biodegradation caused by compost storage affected the thermal properties in a significant manner, as the DSC curves of the first heating run after 8 weeks at 30 °C differed significantly from those after 7 days at 60 °C (see Figure 7), which also occurred for the corresponding transition temperatures and heats of fusions, as is shown in Table 3.

After compost storage for 8 weeks at 30 °C, the glass temperatures of the PLA hard segments *T*_g,hs_ were increased by 4 °C (REF, V1, and V2), 2 °C (V3) and 3 °C (V4), as can be seen in Table 3. This shows that annealing and physical aging of the amorphous PLA phase took place during compost storage. Furthermore, an endothermal peak at 65 °C appeared for all compounds, as is shown in Figure 7a. Given that it did not appear after compost storage at 60 °C (see Figure 7b), it was probably the result of a relaxation peak of the amorphous PLA caused by long storage times below the PLA glass temperature. Given that it was more pronounced for the REF, V1, and V2, one can conclude that the biodegradation had proceeded more significantly.

After compost storage for 7 days at 60 °C (see Table 3), only the V1 and V2 exhibited an increase in the *T*_g,hs_ of 2 °C, whereas it was decreased by 2 °C (REF), 5 °C (V3), and 3 °C (V4). Due to the pronounced state of disintegration, it is unclear if the increase in the *T*_g,hs_ of the V1 and V2 was linked to the PLA or to the PLA being chemically modified by biodegradation. The decreases in the *T*_g,hs_ of the REF, V3, and V4 can be interpreted through the softening of amorphous PLA due to the absorption of water or other low molecular components. In addition, the DSC traces of the REF and V1 to V4 exhibited a second step-like transition at 75 °C (see Figure 7b), whose appearance cannot be explained yet.

The PBAT melting temperature *T*_m1_ of the REF was increased from 113 °C to 117 °C after compost storage for 8 weeks at 30 °C. This shows that post-crystallization processes led to a growth of lamellae thickness. The *T*_m1_ values of the V1 to V4 initially ranged between 105 and 109 °C, but were significantly increased during compost storage: They increased by 16 °C (V1), 11 °C (V2), 9 °C (V3), and 12 °C (V4). The initial mean lamellae thicknesses of the crystalline PBAT of the V1 to V4 were smaller than those of the REF due to the crystallization-disturbing effects of the CECLs. This initial situation allowed for post-crystallization that led to a significant growth of mean lamellae thicknesses that were similar for the REF, V3, and V4 and higher mean growth for the V1 and V2. During compost storage for 8 weeks at 30 °C, the heats of fusion *ΔH*_m1_ increased significantly. For the REF, V1, and V2, they increased by a factor of two. For the V3 they increased by a factor of 1.5, and for the V4, they increased by a factor of 4, which indicated post-crystallization.

The PLA melting temperature *T*_m2_ of the REF increased by 8 °C after compost storage for 8 weeks at 30 °C. This showed that post-crystallization had taken place, as well as a growth of lamellae thickness. For the V1 to V4, the *T*_m2_ decreased between 0 °C and 3 °C, which indicated that the structural changes caused by the CECLs limited the growth of the lamellae thickness. The heats of fusion *ΔH*_m2_ were increased by a factor of 2.5 for the REF, V1, and V3, which indicated post-crystallization in their PLA phases. The *ΔH*_m2_ remained constant for the V2, whereas it decreased by a factor of 0.4 for the V4. When considering the changes in the *ΔH*_m2_, one has to keep in mind that the melting peaks of the PLA overlap partly with those of the PBAT. Thus, the precise determination of the *ΔH*_m2_ requires peak deconvolution, which may lead to smaller differences between the REF and V1 to V4.

After compost storage for 8 weeks at 30 °C, all compounds had higher *T*_m1_, increased *ΔH*_m1_, and partly increased *ΔH*_m2_. This explains the increasingly brittle behavior of the films, the initial increases in Young’s moduli, and their “constancy” during compost storage, in spite of the first biodegradation, as is shown in Figure 5.

After compost storage for 7 days at 60 °C all compounds exhibited higher melting temperatures of the PBAT *T*_m1_—5 °C (REF), 20 °C (V1), 13 °C (V2), 7 °C (V3), and 10 °C (V4)—and increased heats of fusion *ΔH*_m1_—by a factor of three (REF, V1 and V2), factor of two (V3), and factor of four (V4). This indicated pronounced post-crystallization and growth of the lamellae thickness, which can be partly attributed to the biodegradation of the amorphous PBAT [38].

The PLA melting temperature *T*_m2_ of the REF increased by 7 °C after 7 days at 60 °C, which indicated post-crystallization with the formation of thicker lamellae. For the V1 to V4, the *T*_m2_ decreased by 5 °C (V1), 10 °C (V2), 1 °C (V3), and 4 °C (V4). This shows that the CECLs hindered the formation of thick lamellae. The heats of fusion *ΔH*_m2_ increased by a factor of 3 (REF), a factor of 2 (V1), a factor of 1.2 (V2) and a factor of 2.5 (V3), which indicated post-crystallization of the PLA. For the V4, the *ΔH*_m2_ could be considered as unchanged.

The crystallization temperatures *T*_cr_ of the REF, V1, and V2 increased by more than 10 °C for both composting conditions. For the V3 and V4, the *T*_cr_ decreased by 15 °C and 4 °C, respectively, thus indicating structural changes of the polymer chains that hindered crystallization.

Interestingly, the REF, V1, and V2 exhibited enthalpy minima at 90 to 95 °C after compost storage at 30 °C. Therefore, overall enthalpies were determined for the temperature range of 20 to 170 °C, as well as for three temperature ranges, in order to address the disintegration processes of the amorphous PBAT (20 to 95 °C), the crystalline PBAT (95 to 142 °C) and the crystalline PLA (142 to 170 °C). Table 4 reveals that the biodegradation reached different stages in the corresponding temperature ranges. The V3 and V4 were hardly disintegrated according to visual inspection (see Figure 1), and for both storage conditions had a total enthalpy of 22 J/g. Therefore, this value was taken as a reference value for the following considerations. This can also be justified as the overall enthalpies of the REF and V1 to V4, and the initial states were (21 ± 2) J/g in the first run and (17 ±1) J/g in the second run.

After compost storage for 7 days at 60 °C, the total enthalpies of the REF, V1, and V2 were around 33 J/g, whereas they remained around 22 J/g for the V3 and V4, which were identical to the total enthalpies of the REF, V1, V3, and V4 after compost storage for 8 weeks at 30 °C (with the V2 being the only compound with the highest disintegration rate at a total exhibited enthalpy of 26 J/g). In the second heating runs, similar DSC traces with similar mean total enthalpies of (16.2 ± 0.4) J/g for compost storage after 8 weeks at 30 °C and (16.4 ± 1.1) J/g for compost storage of 7 days at 60 °C were measured for the REF and V1 to V4. This means that the difference between the first heating runs could mainly be attributed to processes of biodegradation.

Obviously, the V3 and V4 disintegrated the least and had identical total enthalpies. However, the enthalpies were differently distributed between the temperature ranges for the two conditions of compost storage: between 20 and 95 °C, they were 7 J/g (8 weeks at 30 °C) versus 12 J/g (7 days at 60 °C), and between 95 and 142 °C, they were 12.5 J/g (8 weeks at 30 °C) versus 10 J/g (7 days at 60 °C).

The enthalpies with respect to the temperature ranges indicated that different biodegradation processes occurred at 30 °C and 60 °C. The total enthalpies after compost storage for 7 days at 60 °C of the REF, V1, and V2 were between 31 and 33 J/g. The enthalpies between 20 and 95 °C, as well as between 95 and 142 °C, increased by 60 to 70% with respect to the REF at 30 °C. This shows that new stages of biodegradation were reached in both temperature ranges. The enthalpies between 142 and 170 °C showed that the crystalline PLA seemed to not be subjected to the severe biodegradation at that point.

### 2.5. Effects of Compost Storage on Mean Molecular Masses

**Figure 8:**
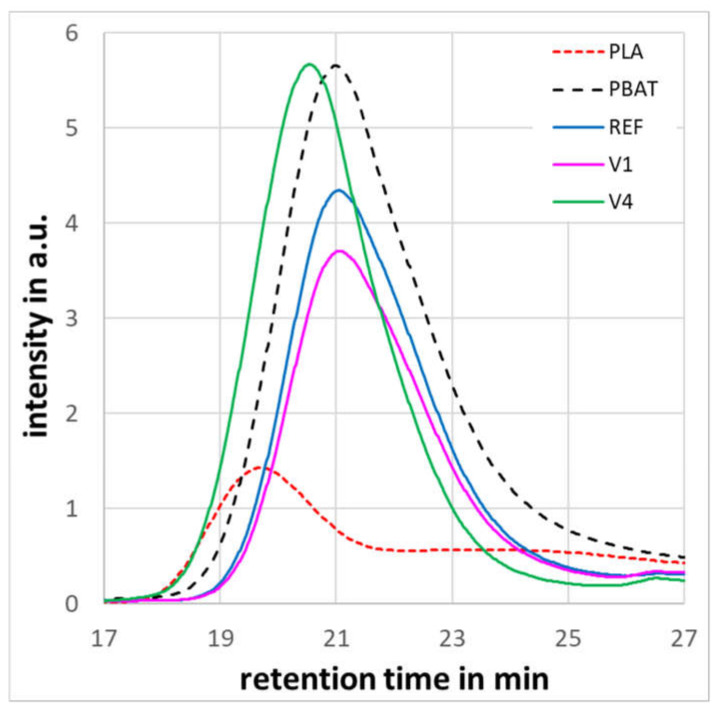
GPC curves of pure PBAT, REF, V1, V4 and pure PLA.

Gel permeation chromatography (GPC) was used to check if molecular degradation has occurred during compost storage, as is shown in Table 5. The results confirm the earlier findings of MFR measurements [33] that the CECLs caused cross-linking in the V3 and V4, as their molecular masses (Mn ≈ 75,000 g/mol) are roughly doubled compared to the REF. The REF, V1, and V2 with molecular masses around 44,000 g/mol showed that the CECLs did not significantly change the molecular masses and, as a consequence, the macromolecular structure. Thus, the CECL molecules have to be either grafted to a polymer chain if there are chemical reactions or remain as low molecular components in the polymer matrix. The significantly higher MFR values of the V1 and V2 when compared to the REF in [33] were explained by chain scission. However, according to the GPC results, as are shown in Table 5, this cannot be the reason for the higher melt flow rate (MFR) values. This means that the unreacted CECLs in the V1 and V2 have to act as flow agents. The GPC curves of the REF and V1 to V4 showed a single peak, as is shown in Figure 8, in contrast to double peak chromatograms observed by Fu et al. [39], which were independent of the PBAT–PLA ratio. The single peak chromatograms can be explained by fact that the height of the pure PBAT peak exceeded by five to six times that of the pure PLA regarding what corresponded to the PBAT–PLA ratio. Thus, the PLA peak remained small and overlaid by the PBAT peak.

Compost storage at 30 °C did not change the molecular mass of the REF, V1, V3, and V4 with respect to the accuracy of the measurement. Only the V2 exhibited further cross-linking with increased molecular masses in the order of 10%. Compost storage at 60 °C led to molecular degradation of the REF and V1 after 7 days, and also to further cross-linking of the V2 in the order of 10%. For the V3 and V4, molecular degradation was neither detected at 30 °C nor at 60 °C.

The GPC results clearly show that, under the given composting conditions, molecular degradation started only after 3 days at 60 °C for the REF and V1. This means that the determined mass losses shown in Figure 2 and the growth of the hole area have to have been caused by mechanical disintegration towards micro-plasticization.

## 3. Discussion

The disintegration behavior of the PBAT/PLA compound M VERA^®^ B5029 was significantly affected by the four multi-functional CECLs when subjected to biodegradation in compost at 60 °C, whereas at 30 °C, only a degradation of the mechanical properties was observed after 4 to 8 weeks. The time dependent mass changes at 60 °C shows that biodegradation starts with a mass increase in the blown films that can be attributed to the absorption of water and other low molecular components originating from the compost as well as microbial settling. Mass losses occur after a certain initiation time and have to exceed 10% to become significant. Thus, the initiation times determined by mass loss can be considered as maximum initiation times. Furthermore, with ongoing disintegration, the probability of mass loss increases due to fracturing, e.g., into micro-plastic particles and their removal during sample cleaning.

The mean masses determined by GPC show that molecular degradation from an *M*_n_ of 43,000 g/mol to 35,000 g/mol only occurred for the REF and V1 after compost storage for 7 days at 60 °C. This means that the observed mass loss and structural disintegration were not due to molecular degradation but were mainly due to mechanical disintegration. This finding justifies the single process kinetic analysis via the time dependent increase of the cross-sectional hole area as a measure of disintegration, which is characterized by two quantities—initiation time and disintegration time. The initiation time at least has to depend on sample thickness, as mechanical disintegration and microbial attacks start at the surface with time proportional amounts of disintegration.

In comparison to the REF, the CECLs led to expedited disintegration of the V1 and V2 during compost storage at 60 °C. Given that the V1 exhibited molecular degradation and the V2 did not, mass loss and increasing cross-sectional hole areas have to have been the result of mechanical disintegration by fracturing towards micro-particles that remained in the compost or were lost during sample cleaning. The mechanical properties of elongation at break and tensile strength seemed to be very sensitive to structural changes prior to visible mechanical disintegration. This is stated by the fact that the V3 and V4 that were cross-linked by the CECLs exhibited reduced elongations at break and tensile strengths after compost storage for 4 to 8 weeks at 30 °C, in spite of no visible disintegration.

After compost storage, a remarkable amount of low molecular components was found in the PBAT/PLA films (see Table 1), which caused a swelling of the films on one side and promoted annealing and post-crystallization on the other side. Both processes may have propagated mechanical disintegration due to stress cracking. Given that the PBAT/PLA films have anisotropic morphologies with different properties in the ED and TD, it is probable that the corresponding directions of crack propagations were anisotropic. This explains why different time dependent decays of the mechanical properties of elongation at break and tensile strength were found. Direct comparison with the results of other researchers could not be made due to the lack of literature correlating the disintegration behavior with mechanical properties and the conjugated effects of the CECLs and processing.

It seems that mechanical disintegration dominated the early stages of the biodegradation of the investigated PBAT/PLA films. This means that the films were first fractured to micro-particles. Such behavior was also found by Fu et al. [40] for PBAT and PLA during freshwater degradation. Due to the increased surface, the diffusion of low molecular components causing, e.g., hydrolytic degradation of polymer chains, in these micro-particles was eased, as was microbial attack, from the surface. However, only after undergoing a sufficient mechanical disintegration to micro-particles, a noteworthy molecular degradation of polymer chains can occur with subsequent metabolization by compost microbes.

Due to the environmental and temperature conditions during compost storage, morphological changes may have occurred due to annealing of the amorphous phases and post-crystallization with the growth of lamellae thickness, as well as the formation of new lamellae combined with the dissolving of thin lamellae. All these processes reduce the free volume of the PBAT/PLA films and may increase interlamellar internal stresses, as the crystalline lamellae partly hinder free shrinkage if inclined to one another. If low molecular components diffuse into the films, the internal stresses are released faster at sites of stress concentrations and form cracks and voids as the beginnings of mechanical disintegration. The rate of crack formation and propagation depends on the toughness of the PBAT/PLA compounds, and, thus, on their molecular masses and crystallinity. Therefore, the mechanical degradation of the V3 and V4 was reduced when compared to the REF, V1, and V2 because of their almost doubled molecular masses, which correspondingly reduced the diffusion of low molecular components and conserved toughness for a longer time.

Interestingly, carbodiimide-based CECLs were used for the V3 and V4, which are commercially sold as hydrolysis stabilizers for, e.g., PLA. Investigations of the anti-hydrolytic effects of carbodiimides on PLA [34] and PLA wood flour composites [35] showed that the molecular masses were not decreased by processing after an addition of more than 0.5 wt.%. It was possible to maintain the molecular masses on the level of neat PLA. From this fact, one can conclude that the almost doubled molecular masses of the V3 and V4 have to have been attributed only to cross-linking in the PBAT phase.

It is obvious that the disintegration processes occurring in the PBAT/PLA films are complex and act on different time scales with respect to properties such as mass loss, tensile strength, elongation at break, transition temperatures, and heats of fusion. If the blown films are subjected to compost storage, the first processes are mainly water diffusion in the amorphous phases of the PBAT and PLA and the settling of micro-organisms on the film surfaces. As a compost consists of a manifold of micro-organisms, other substances of low molecular weights—being yet unknown—can diffuse in the available free volume of water-softened amorphous phases, where they undergo further chemical reactions and initiate stress cracking. This propagates mechanical disintegration by micro-void formation and fracturing. As fracturing is a defect-controlled process, elongations at break and tensile strengths can already decrease if there are small void concentrations close to the surface at the early stages of biodegradation. Internal stress fields in the films (what is probable due to the anisotropic structure generated by the film blowing process), propagate these voids and ease the fracturing of the films to micro-plastic particles. With respect to complete degradation, this fracturing towards micro-plastic particles is beneficial. Firstly, the diffusion paths for low molecular weight components (such as water) decrease, and hydrolytic molecular chain degradation requires shorter initiation times. Secondly, the fracturing generates a new surface area where micro-organisms can settle and metabolize the polymer.

## 4. Materials and Methods

### 4.1. Materials and Sample Preparation

Four chain extending cross-linkers (1 wt.%) were compounded to the reference PBAT/PLA blend (REF) M·VERA^®^ B5029 [40] from BIO-FED, a branch of AKRO-PLASTIC GmbH, Cologne, Germany. M·VERA^®^ B5029 consists of 65% PBAT (matrix), and 11% PLA (disperse phase) and 24% of CaCO_3_ filler particles. It is mainly used for packaging and agricultural applications. The following CECLs were employed:

- V1—tris(2,4-di-tert-butylphenyl)phosphite, Songnox 1680 (Songwon Industrial Co, Ulsan, South Korea) [41],
- V2—1,3-phenylenebisoxazoline, 1,3-PBO powder (Evonik, Essen, Germany) [42],
- V3—aromatic polycarbodiimide, Stabaxol P110 (Lanxess, Cologne, Germany) [43], and
- V4—poly(4,4-dicyclohexylmethane carbodiimide), Carbodilite HMV-15CA (Nisshinbo, Tokyo, Japan) [44].

All ingredients were evenly mixed using a Mixaco CM 150-D (Mixaco Maschinenbau, Neuenrade, Germany) and compounded by a twin-screw extruder (FEL 26 MTS, Feddem GmbH, Sinzig, Germany) with 26 L/D, screw speed of 260 rpm, and output rate of 20 kg h^−1^. The films were manufactured using the blow molding machine LF-400, (Labtech Engineering Company Co., Ltd., Samut Prakan, Thailand) with an extrusion temperature of 165 °C and a blow-up ratio (BUR) of 1:2.5 for 25 µm-thick films. This was to affirm almost similar morphology developments in all investigated compounds. CECLs influence the melt flow ratio and, thus, affect the melt viscosities of the compounds [33]. For the given blowing condition, this may affect film thicknesses and draw ratios (DR), as is shown in Table 6. The film thicknesses (Table 6) exhibited relatively small standard deviations, because they were determined on stripes before compost storage from a relatively short section of blown films. A device for inline-monitoring of the thickness was not available.

The film thicknesses during blown film extrusion typically may vary by up to 15% [45,46] for both the ED and TD due to process fluctuations such as changes in melt viscosity due to variations in die temperature or die gap control. Since the overall mean thickness in Table 6 was (28 ± 2) µm, the variation range was within the limit.

The samples were stabilized at 23 °C for 24 h after extrusion due to customers’ requirements enabling further packaging processing. Films were cut to tensile test stripes with the dimensions 170 × 15 mm and weighed. Trays with at least 3 L of volume were filled with a compost (Plantiflor Pro Natur torffrei, Dortmund, Germany) with 3 subsequent layers: a bottom layer of compost (thickness 5 cm), a layer of individualized test specimens, and a top layer of compost (thickness > 5 cm). To prevent cross-contamination, differently colored trays were used for the REF and V1 to V4 and stored in an oven at 30 °C or 60 °C, respectively, with 60% relative humidity to be close to industrial composting conditions described by ISO 16926:2018. The storage times at 30 °C were 168, 336, 672 and 1344 h; at 60 °C they were 24, 48, 72, 96, 120, 144 and 168 h. Since the V3 and V4 hardly showed visual disintegration, they were also subjected to compost storage at 60 °C for 336 and 672 h. To follow ISO 16926:2018, pH values were determined when the samples were removed and cleaned.

### 4.2. Methods

#### 4.2.1. Determination of Mass Change after Disintegration in Compost

The film masses after compost exposure were measured using the Sartorius BP221S balance (Sartorius AG, Göttingen, Germany) with a measuring accuracy of 0.1 mg. The determined masses showed scatters in the range of 3 to 7% due to sticking compost particles, absorbed disintegration substances, and loss of micro-plastic particles during cleaning in cases of severe film disintegration. For better comparison of mass changes of the REF and V1 to V4, the data were converted to relative masses:

(1) $$m_{rel}\left(t\right)=\frac{m\left(t\right)}{m_{0}}$$

with mass *m(t)* after the given storage time in compost and initial mass *m*_0_.

#### 4.2.2. Kinetics of the Process of Disintegration in Compost Due to the Decrease in Film Cross-Section

After the given storage times, the samples were taken out of the compost, cleaned with a soft paint brush to remove compost particles, and subsequently stored at 23 °C/50% r.h. Then pictures of the PBAT/PLA films were taken with a Sony Cyber-shot DSC-HX60V camera.

The sample films appeared in greyish colors, whereas holes appeared in black. Then, the pictures were discriminated to black and white pixels, and the corresponding pixel areas were calculated using the public domain image analysis software Fiji [47]. The degree of disintegration *x_disint_* was determined by

(2) $$x_{disint}\left(t\right)=\frac{A_{black}\left(t\right)}{A_{white}\left(t\right)+A_{black}\left(t\right)}$$

with the black pixel area represented as *A_black_* and white pixel area represented as *A_white_*.

The quantitative kinetic approach assumes that the micro-organisms from the compost attack the film at the contact areas and start disintegration processes from the surface. Since the compost has a coarse and crumbly structure, the disintegration does not happent uniformly on the surface but locally at certain points. After an initiation time *t_init_*, first, holes appear in the films, which grow in size and number, and, thus, decrease the remaining film cross-section. If one assumes that the time dependent change of the film cross-section $\frac{dA^{film}}{dt}$ depends on the available film cross-section $A^{film}$, one gets

(3) $$\frac{dA^{film}}{dt}\sim \;A^{film}\left(t\right)=A_{0}-A^{hole}\left(t\right)$$

with hole area $A^{hole}$.

After dividing the initial cross-section *A*_0_, one gets the normalized rate equation with the temperature dependent disintegration constant $k_{disint}$:

(4) $$\frac{dA_{rel}^{film}}{dt}=-k_{disint}\left(T\right)\;A_{rel}^{film}\left(t\right)$$

Solving by separation of variables and subsequent integration over equivalent limits gives:

(5) $$ln\;A_{rel}^{film}\left(t\right)=-k_{disint}\left(T\right)\;t+C$$

with the integration constant *C* = 0.

Solving for $A_{rel}^{film}\left(t\right)$ and substitution by $x_{disint}\left(t\right)$ yields:

(6) $$x_{disint}\left(t\right)=1-e^{-k_{disint}\left(T\right)\;t}$$

To take into account the initiation time *t_i_*_nit_ of the disintegration process, one has to modify Equation (6) into:

(7) $$x_{disint}\left(t\right)=1-e^{-k_{disint}\left(T\right)\;\left(t-t_{init}\right)}$$

or

(8) $$x_{disint}\left(t\right)=1-e^{-\frac{\left(t-t_{init}\right)}{\tau_{disint}}}$$

with the disintegration time *τ_disint_* being a measure of how fast the disintegration process proceeds.

A least square fitting procedure for Equation (8) with the excel solver was used to determine *t_init_* and *τ_disint_* to quantify the disintegration kinetics.

#### 4.2.3. Tensile Tests and Mechanical Properties

Young’s modulus, tensile strength, and fracture strain were determined according to ISO 527-3 using a tensile testing machine (2.5 kN Zwicki, Zwick Roell, Ulm, Germany) at 23 °C/50% r.h. and a crosshead speed of 200 mm min^−1^. Tensile tests were performed in the extrusion direction (ED) and transversal direction (TD) with n = 5 as long as the sample disintegration allowed that. All samples having initial dimensions of 170 × 15 mm were conditioned at 23 °C and 50% r.h. for 24 h before testing to adjust the same testing conditions under which the initial properties were determined in [33].

#### 4.2.4. Differential Scanning Calorimetry (DSC) and Thermal Properties

DSC experiments were performed using a DSC Diamond (Mettler Toledo, Greifensee, Switzerland) for granules and a DSC 214 Polyma (Netzsch Gerätebau GmbH, Selb, Germany) for films in standard Al pans with pinholed lids in three steps—1st heating, cooling, 2nd heating. The measuring conditions were:

- Sample weight *m_s_*: (6 ± 1) mg
- Starting temperature *T_start_*: 0 °C
- End temperature *T_end_*: 200 °C
- Heating/cooling rate: 10 K min^−1^
- Repetition: n ≥ 2

The DSC traces according to ISO 11357-3:2018 were evaluated with respect to glass transition temperatures of the hard segment *T*_g,hs_, melting temperatures of PBAT *T_m1_* and PLA *T_m2_* and the corresponding heats of fusion *ΔH*_m1_, *ΔH*_m2_ (1st run). Crystallization temperatures *T*_cr_ and heats of crystallization *ΔH*_cr_ were determined from the cooling run. All samples were also conditioned at 23 °C and 50% r.h. for 24 h before testing.

To estimate the content of humidity, the enthalpies between 70 and 90 °C were determined from the 1st and 2nd run. Finally, the film masses after the 2nd run were measured to determine the mass loss due to all low molecular weight components.

#### 4.2.5. Gel Permeation Chromatography (GPC)

The granules of pure PBAT and PLA as well as film samples of the REF and V1 to V4 were dissolved in THF and filtered using PTFE filters with a mesh of 0.45 µm into vials of 2 mL. All samples showed a turbidity due to a filler content of 24%. The sample concentration in each vial was 2.5 g/L.

The GPC measurements were conducted by a Waters HPLC system equipped with a Waters model e2695 and Waters model 2414 differential refractometer (Waters Corporation, Massachusetts United States of America). The GPC system was calibrated with polystyrene standards having molecular masses of 580; 10,440; 38,640; 132,900; 492,500; and 990,500 g/mol.

The measuring conditions were:

- Series of gel-mixed bed columns: PL gel MIXED-A (300 × 7.5 mm, 20 µm) + PL gel MIXED-B (300 × 7.5 mm, 10 µm) + PL gel MIXED-D (300 × 75 mm, 5 µm);
- Mobile phase: tetrahydrofuran (THF) stabilized with butylated hydroxytoluene;
- Temperature: 40 °C;
- Injection volume: 100 µL;
- Flow rate: 1 mL/min;
- Detector: refractive index detector (RI).

All data processing was carried out using Empower 3 software (Version FR 3).

## Figures and Tables

**Table 1:** Effects of conditions of compost storage on mass loss and relative mass loss after second run, and on enthalpies from 70 to 90 °C of first and second run.

Storage	8 Weeks at 30 °C			7 Days at 60 °C		
	Δ*H* between 70 to 90 °C	Mass Loss	Relative Mass Loss	Δ*H* between 70 to 90 °C	Mass Loss	Relative Mass Loss
Unit	mJ	mg	-	mJ	mg	-
REF	15	0.25	0.04	34	0.43	0.07
V1	21	0.44	0.07	46	0.67	0.11
V2	23	0.45	0.08	45	0.74	0.12
V3	26	0.24	0.04	28	0.25	0.04
V4	27	0.20	0.03	25	0.13	0.02

**Table 2:** Kinetic parameters of REF and V1 to V3 compounds over 7 days at 60 °C of disintegration in compost.

Kinetic Parameters		Compound			
	Unit	REF	V1	V2	V3
Time *t*_80%_ to 80% of initial mass	h	132.7	105.6	105.8	174.1
Initiation time *t*_init_	h	32.8	34.1	23.7	45.8
Disintegration time *τ*_disint_	h	385.1	147.3	94.8	1179.5
R^2^ of fits		0.96	0.95	0.97	0.83

**Table 3:** Effects of compost storage (8 weeks/30 °C and 7 days/60 °C) on glass temperature of hard segments *T*_g,hs_, melting temperatures *T*_m1_ of PBAT, and *T*_m2_ of PLA with corresponding heats of fusion *ΔH*_m1_ and *ΔH*_m2_ determined from first heating and with crystallization temperature *T*_cr_ and crystallization enthalpy *ΔH*_cr_ determined from cooling run of REF and V1 to V4; accuracy of temperatures is ±1 °C; data before compost storage were taken from [33].

Compound	*T*_g,hs_	*T*_m1_	*ΔH*_m1_	*T*_m2_	*ΔH*_m2_	*T*_cr_	*ΔH*_cr_
	°C	°C	J/g	°C	J/g	°C	J/g
	midpoints	PBAT	PBAT	PLA	PLA		
Before storage in compost							
REF	50	113	5.4 ± 0.4	150	1.1 ± 0.1	75	15.2 ± 0.9
V1	48	106	5.4 ± 0.5	152	1.2 ± 0.1	73	15.1 ± 0.1
V2	49	109	5.1 ± 0.2	153	2.9 ± 0.1	72	14.7 ± 0.1
V3	50	108	6.4 ± 0.1	150	1.2 ± 0.1	76	11.7 ± 0.3
V4	49	105	3.7 ± 0.1	153	3.5 ± 0.1	75	12.7 ± 0.6
After 8 weeks at 30 °C							
REF	53	117	9.9 ± 0.1	158	2.8 ± 0.2	87	11.8 ± 0.1
V1	52	122	10.2 ± 0.4	151	3.4 ± 0.3	83	12.0 ± 0.2
V2	53	120	10.4 ± 0.1	150	2.6 ± 0.1	82	12.1 ± 0.4
V3	52	117	9.2 ± 0.1	150	2.8 ± 0.1	60	12.6 ± 0.1
V4	52	118	13.3 ± 0.2	151	1.4 ± 0.1	71	11.7 ± 0.2
After 7 days at 60 °C							
REF	48	119	15.8 ± 0.3	157	3.1 ± 0.1	93	11.1 ± 0.2
V1	50	126	18.7 ± 0.7	147	2.4 ± 0.8	88	10.8 ± 1.5
V2	51	123	16.4 ± 0.1	143	3.4 ± 0.3	84	11.8 ± 0.4
V3	45	117	13.3 ± 0.2	149	2.8 ± 0.1	61	11.7 ± 0.2
V4	46	115	13.0 ± 0.6	149	2.9 ± 0.1	71	11.4 ± 0.9

**Table 4:** Enthalpies of REF and V1 to V4 between 20 to 170 °C to differentiate states of biodegradation of amorphous PBAT, crystalline PBAT, and crystalline PLA; standard deviation of enthalpy: ±1 J/g.

	Temperature Range in °C				
	20 to 170	20 to 95	95 to 142	142 to 170	20 to 170
Compound	Enthalpy after Storage for 8 Weeks at 30 °C in J/g				2nd Run
REF	22	7 *	11 *	3	16
V1	22	9	10	3	17
V2	26	11	12	3	17
V3	22	7	13	2	16
V4	22	7	12	3	16
	Enthalpy after Storage for 7 Days at 60 °C in J/g				2nd Run
REF	33	12 *	17 *	4	18
V1	33	13	17	3	15
V2	31	13	16	2	16
V3	22	10	10	2	17
V4	22	11	10	1	17

* Temperature intervals for REF were 20 to 90 °C and 90 to 142 °C.

**Table 5:** Determination of the mean molecular masses due to GPC measurements with number-average molecular mass *M*_n_, weight-average molecular mass *M*_w_, and polydispersity index of the polymer PDI; accuracy of measurements is 2000 g/mol for *M*_n_ and 3000 g/mol for *M*_w_.

Compound	*M*_n_	*M*_w_	PDI
	g/mol	g/mol	-
Initial state			
PBAT	43,000	107,000	2.45
REF	44,000	83,000	1.89
V1	42,000	81,000	1.92
V2	46,000	92,000	1.98
V3	75,000	149,000	1.99
V4	72,000	141,000	1.95
PLA	218,000	309,000	1.42
After compost storage for 2 weeks at 30 °C			
REF	46,000	86,000	1.89
V1	47,000	87,000	1.88
V2	52,000	105,000	1.99
V3	75,000	160,000	2.12
V4	75,000	148,000	1.98
After compost storage for 8 weeks at 30 °C			
REF	43,000	82,000	1.94
V1	43,000	83,000	1.92
V2	51,000	103,000	2.00
V3	78,000	162,000	2.07
V4	74,000	148,000	2.01
After compost storage for 3 days at 60 °C			
REF	41,000	80,000	1.95
V1	42,000	79,000	1.90
V2	52,000	99,000	1.91
V3	72,000	154,000	2.14
V4	71,000	141,000	1.98
After compost storage for 7 days at 60 °C			
REF	34,000	67,000	1.97
V1	37,000	71,000	1.93
V2	49,000	96,000	1.97
V3	72,000	154,000	2.13
V4	72,000	143,000	1.99

**Table 6:** Mean thicknesses of films of the reference (REF) and CECL-modified (V1 to V4) PBAT/PLA blends and corresponding draw ratio.

	Thickness in µm	Draw Ratio
REF	25.8 ± 0.7	12.4
V1	30.5 ± 0.5	10.5
V2	26.3 ± 0.5	12.2
V3	27.3 ± 0.6	11.7
V4	29.9 ± 0.8	10.7

Note: Thicknesses were determined on stripes before compost storage.

## Data Availability

Datasets analyzed and generated during the study will be provided upon request.
